# NIR‐Triggered Phototherapy and Immunotherapy via an Antigen‐Capturing Nanoplatform for Metastatic Cancer Treatment

**DOI:** 10.1002/advs.201802157

**Published:** 2019-03-13

**Authors:** Meng Wang, Jun Song, Feifan Zhou, Ashley R. Hoover, Cynthia Murray, Benqing Zhou, Lu Wang, Junle Qu, Wei R. Chen

**Affiliations:** ^1^ Key Laboratory of Optoelectronic Devices and Systems of Ministry of Education and Guangdong Province College of Optoelectronic Engineering Shenzhen University Shenzhen 518060 P. R. China; ^2^ Center of Interdisciplinary Biomedical Education and Research College of Mathematics and Science University of Central Oklahoma OK 73034 USA

**Keywords:** antigen capture, immunotherapy, metastatic cancer, phototherapy, upconversion

## Abstract

Combined phototherapy and immunotherapy demonstrates strong potential in the treatment of metastatic cancers. An upconversion nanoparticle (UCNP) based antigen‐capturing nanoplatform is designed to synergize phototherapies and immunotherapy. In particular, this nanoplatform is constructed via self‐assembly of DSPE‐PEG‐maleimide and indocyanine green (ICG) onto UCNPs, followed by loading of the photosensitizer rose bengal (RB). ICG significantly enhances the RB‐based photodynamic therapy efficiency of UCNP/ICG/RB‐mal upon activation by a near‐infrared (NIR) laser, simultaneously achieving selective photothermal therapy. Most importantly, tumor‐derived protein antigens, arising from phototherapy‐treated tumor cells, can be captured and retained in situ, due to the functionality of maleimide, which further enhance the tumor antigen uptake and presentation by antigen‐presenting cells. The synergized photothermal, photodynamic, and immunological effects using light‐activated UCNP/ICG/RB‐mal induces a tumor‐specific immune response. In the experiments, intratumoral administration of UCNP/ICG/RB‐mal, followed by noninvasive irradiation with an NIR laser, destroys primary tumors and inhibits untreated distant tumors, using a poorly immunogenic, highly metastatic 4T1 mammary tumor model. With the simultaneous use of anti‐CTLA‐4, about 84% of the treated tumor‐bearing mice achieve long‐term survival and 34% of mice develop tumor‐specific immunity. Overall, this antigen‐capturing nanoplatform provides a promising approach for the treatment of metastatic cancers.

## 1 Introduction

Cancer immunotherapy has emerged as a promising cancer therapeutic modality for recurrent or metastasized cancer.^1^ Over the past few years, significant effects have been reported in treating metastatic tumors via activation of the host immune system, using cytokine therapy,^2^ immune checkpoint blockade therapy,^3^, ^4^ and adoptive cell therapy.^5^, ^6^ Although significant advances have thus been achieved in cancer immunotherapy, it still suffers from limitations, such as individual variations, low therapeutic responses, and severe adverse effects.^7^, ^8^, ^9^ Despite these efforts, an approach that can increase the immune response rate, selectively destroy primary solid tumors, and eliminate metastatic lesions is highly desirable.

Phototherapy, particularly photodynamic therapy (PDT) and photothermal therapy (PTT), has gained increasing acceptance as a noninvasive medical technique for the local treatment of a variety of cancers, due to its favorable therapeutic efficacy.^10^, ^11^, ^12^, ^13^ Moreover, phototherapy has the potential to induce antitumor immunity to kill residual or metastatic tumor cells.^14^, ^15^, ^16^ Several studies have demonstrated that phototherapy can elicit immunogenic cell death (ICD) by inducing dying tumor cells to release tumor‐derived protein antigens (TDPAs), such as damage‐associated molecular patterns and neoantigens.^17^, ^18^, ^19^, ^20^ TDPAs act as potent danger signals that induce a series of receptor expressions by dendritic cells (DCs), thus stimulating a Th1‐biased immune response. However, the immune response induced by phototherapy is relatively weak and short‐lived. Therefore, a combination of phototherapy with immune therapeutic approaches offers a promising method for cancer treatment.

Conventional phototherapy still faces limitations such as light tissue penetration depth and direct stimulation of the host immune system.^21^, ^22^, ^23^ Recently, various nanomaterials (e.g., metal–organic frameworks, noble metal nanoparticles, and organic nanocarriers) have been used to achieve near‐infrared (NIR) light or X‐ray triggered phototherapy to improve phototherapeutic outcomes.^24^, ^25^, ^26^, ^27^, ^28^ Among these nanomaterials, due to their ability to convert NIR light to visible or ultraviolet light, upconversion nanoparticles (UCNPs) have been extensively used as NIR‐triggered mediators for both PTT and PDT.^29^, ^30^, ^31^, ^32^, ^33^, ^34^, ^35^, ^36^ Moreover, combined with immune therapeutic approaches, UCNP‐based phototherapy has been reported to achieve an effective antitumor response.^37^, ^38^ Due to inefficient presentation of the tumor antigen, most photo‐immunotherapeutic approaches require further strengthening of the antitumor immune response by introducing extraneous adjuvants, which have the potential to cause adverse effects.^39^, ^40^ In addition, UCNP‐based phototherapy usually results in limited stimulation of the host immune system. Therefore, it is imperative to develop a safe and efficient photo‐immunotherapeutic approach for metastasis tumor treatment.

**Figure 1:**
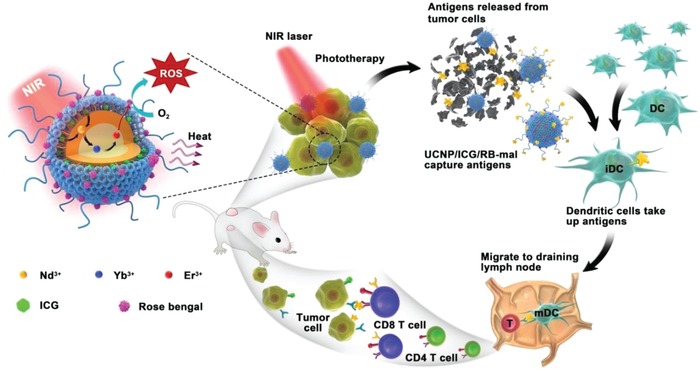
Schematic illustration of both fabrication and mechanism of near‐infrared (NIR)‐triggered antigen‐capturing nanoplatform for synergistic photo‐immunotherapy. Here, the NIR‐triggered antigen‐capturing nanoplatform was constructed via self‐assembly of DSPE‐PEG‐mal and indocyanine green (ICG) onto the oleate‐capped UCNPs, followed by remote loading of rose bengal (RB). Upon NIR laser activation, the photodynamic therapy efficiency of UCNP/ICG/RB‐mal was significantly enhanced by ICG modification, while simultaneously achieving selective photothermal therapy. Next, tumor‐derived protein antigens (TDPAs) arising from UCNP/ICG/RB‐mal based phototherapy can be captured and retained in situ, which increases the effects of antigen uptake by antigen‐presenting cells (APCs) to induce a tumor‐specific immune response. Thus, UCNP/ICG/RB‐mal based phototherapy not only destroys the primary tumors but also inhibits untreated distant tumors via systemic antitumor immune responses.

An NIR‐triggered antigen‐capturing nanoplatform was designed and synthesized, using UCNP as a carrier, indocyanine green (ICG) as a light absorber, rose bengal (RB) as a photosensitizer, and a lipid molecule (DSPE‐PEG‐mal) as an antigen‐capturing agent, for synergistic photo‐immunotherapy (**Figure** **1**). Benefiting from the large absorption cross section of ICG, UCNP/ICG/RB‐mal generated significant ^1^O_2_ and heat to improve phototherapy under NIR laser irradiation and thus enhanced phototherapy elicited ICD with improved release of TDPAs. Furthermore, TDPAs could be captured and retained in situ by UCNP/ICG/RB‐mal, which increases the effects of antigen uptake by antigen‐presenting cells (APCs) and induces a tumor‐specific immune response. This nanoplatform was characterized and its immunological functions were investigated both in vitro and in vivo. Furthermore, the systemic antitumor immunity induced by the UCNP/ICG/RB‐mal nanoplatform was studied under NIR laser irradiation. The obtained results indicate that this novel nanoplatform could be used as therapeutic agent for nanotechnology‐based photo‐immunotherapy for the treatment of metastasis cancer.

## 2 Result and Discussion

### 2.1 Synthesis and Characterization of UCNP/ICG/RB‐Mal

**Figure 2:**
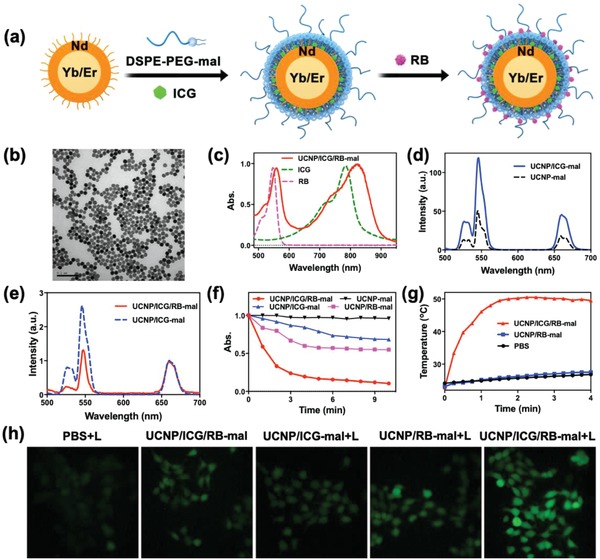
Synthesis and characterization of UCNP/ICG/RB‐mal. a) Schematic drawing showing the fabrication process of UCNP/ICG/RB‐mal. b) TEM image of UCNP/ICG/RB‐mal. c) Vis–NIR absorbance spectra of ICG, RB, and UCNP/ICG/RB‐mal. d) UCL spectra of UCNP‐mal and UCNP/ICG‐mal recorded at the same UCNPs concentration. e) UCL spectra of UCNP/ICG‐mal and UCNP/ICG/RB‐mal. Please note that the UCL spectra were normalized at 660 nm. f) Time‐dependent bleaching of DPBF caused by ^1^O_2_ generation in the presence of 0.5 mg mL^−1^ of UCNP/ICG/RB‐mal under 805 nm laser irradiation. All absorbances were normalized at the zero point of irradiation time. g) Temperature elevations of PBS, UCNP/RB‐mal, and UCNP/ICG/RB‐mal at a concentration of 0.5 mg mL^−1^ under 4 min irradiation. h) Fluorescent images of ROS generation in 4T1 tumor cells.

Synthesis of UCNP/ICG/RB‐mal was achieved with a stepwise method, which is illustrated in **Figure** **2**a. First, oleate‐capped NaYF_4_:Yb/Er@NaYF_4_:Nd core/shell UCNPs were prepared via high‐temperature coprecipitation. Both the crystalline nature and hexagonal‐phased structure of the prepared nanoparticles were confirmed via high‐resolution transmission electron microscopy (TEM) images and powder X‐ray diffraction (XRD) patterns (Figure S1, Supporting Information). To obtain hydrophilic UCNPs, a self‐assembled asymmetric lipid layer, which contains ICG, and DSPE‐PEG‐mal was capped on oleate‐capped UCNPs via hydrophobic/hydrophobic interactions. Then, RB was further encapsulated within UCNP/ICG‐mal via hydrophobic interaction. The resulting UCNP/ICG/RB‐mal exhibited excellent water solubility and inherited the specific morphology of oleate‐capped UCNPs (Figure 2b). The average diameter of UCNP/ICG/RB‐mal measured via dynamic light scattering (DLS) was 41 ± 1 nm (Figure S2, Supporting Information). As shown in Figure 2c, the vis–NIR absorption peaks of UCNP/ICG/RB‐mal are similar to those of ICG and RB, indicating that ICG and RB were successfully loaded into UCNP. The loading capacities of ICG and RB were 1.2 and 0.8 wt%, determined via vis–NIR absorbance spectra (Figure S3, Supporting Information).

ICG have a large absorption cross section (≈30 000 times larger than that of Nd^3+^) and the emission band overlaps well with the Nd^3+ 4^S_3/2_ → ^4^I_9/2_ absorption peaks of UCNPs. Upon excitation at 805 nm, both UCNP‐mal and UCNP/ICG‐mal exhibited sharp and characteristic emission peaks at 521, 541, and 654 nm, which can be assigned to the ^2^H_11/2_, ^4^S_3/2_, and ^4^F_9/2_ → ^4^I_15/2_ transitions of Er^3+^, respectively (Figure 2d). Compared to UCNP‐mal, the upconversion luminescence (UCL) intensity of UCNP/ICG‐mal showed a twofold enhancement (Figure 2d). Both the vis–NIR absorption spectrum and emission spectrum showed an overlap between the RB absorbance band and the green emission peaks of UCNP (Figure S4, Supporting Information). Consequently, the UCL intensity at the green band of UCNP/ICG/RB‐mal decreased markedly compared to that of UCNP/ICG‐mal (Figure 2e). Furthermore, due to energy transfer to RB molecules, ^1^O_2_ production increased when UCNP/ICG/RB‐mal was irradiated with an 805 nm laser, through the bleaching of 1,3‐diphenylisobenzonfuran (DPBF) at 420 nm. Specifically, the DPBF intensity decreased to below 10% within 10 min in the presence of UCNP/ICG/RB‐mal under irradiation at 805 nm laser (0.75 W cm^−2^, NPs concentration: 500 µg mL^−1^), as shown in Figure 2f. Furthermore, increased ^1^O_2_ production was observed in 4T1 cells after incubation with UCNP/ICG/RB‐mal, followed by an 805 nm laser irradiation (3 min, 0.75 W cm^−2^), as measured through the fluorescence emission of the reactive oxygen species (ROS) probe (DCFH‐DA) (Figure 2h). UCNP/ICG/RB‐mal (NPs concentration: 500 µg mL^−1^, 200 µL) experienced a strong temperature increase of up to 50.5 °C at *t* = 90 s under an 805 nm laser irradiation (0.75 W cm^−2^), while no significant temperature increase was detected in UCNPs/RB‐mal and PBS under the same conditions (Figure 2g). Infrared thermal images were also acquired to verify the UCNP/ICG/RB‐mal + laser (L) induced temperature increase (Figure S5, Supporting Information).

### 2.2 In Vitro Immunogenic Cell Death Induced by Phototherapy

**Figure 3:**
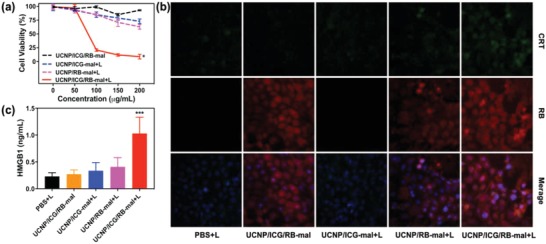
In vitro immunogenic cell death induced via phototherapy. a) Viability of 4T1 tumor cells incubated with different nanoparticles, followed by irradiation with an 805 nm laser (5 min, 0.75 W cm^−2^). (*P < 0.05 vs UCNP/ICG/RB‐mal group.) b) Fluorescent imaging of CRT exposed on the surface of 4T1 tumor cells after different treatments in (a). c) Detection of extracellular release of HMGB1 of 4T1 tumor cells after different treatments in (a). (***P < 0.001 vs PBS + L group.) Data are expressed as mean ± SD (*n* = 4).

The cell viability was determined after treatment by UCNP/ICG/RB‐mal + L (**Figure** **3**a). UCNP/ICG/RB‐mal + L resulted in 91% cell death at a UCNP/ICG/RB‐mal dose of 200 µg mL^−1^ and a light dose of 5 min, 0.75 W cm^−2^. In contrast, no obvious cytotoxicity was observed without light irradiation. The 4T1 tumor killing effect of UCNP/ICG/RB‐mal could be further verified via cell fluorescence images (Figure S6, Supporting Information). Then, the calreticulin (CRT) exposure of treated 4T1 tumor cells was evaluated. As shown in Figure 3b, compared to other control groups, stronger green fluorescence was observed in 4T1 tumor cells in the UCNP/ICG/RB‐mal + L group, suggesting that UCNP/ICG/RB‐mal + L strongly induced CRT exposure on 4T1 tumor cells. Similarly, UCNP/ICG/RB‐mal + L induced the highest level of high‐mobility group box 1 (HMGB1) release from 4T1 tumor cells, as shown in Figure 3c. These results demonstrate that UCNP/ICG/RB‐mal based phototherapy could significantly kill 4T1 tumor cells while enhancing the release of TDPAs.

### 2.3 In Vitro Antigen Capturing and Immune Stimulation Effect of UCNP/ICG/RB‐Mal

**Figure 4:**
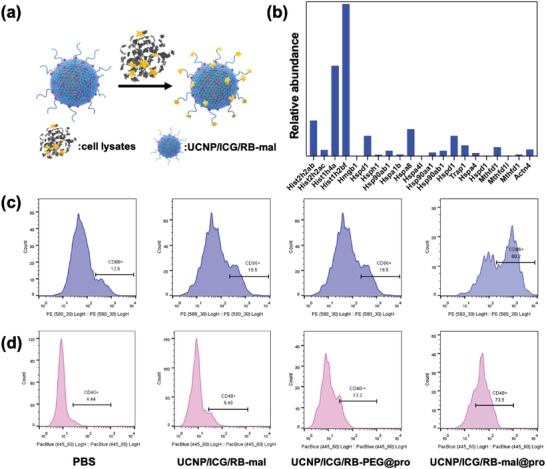
In vitro antigen capturing and immune stimulation effect of UCNP/ICG/RB‐mal. a) Schematic depiction of antigen capture of UCNP/ICG/RB‐mal. b) Relative abundance of TDPAs captured via UCNP/ICG/RB‐mal. c,d) Quantification of CD86^+^ and CD40^+^ expression via flow cytometry for various samples in the in vitro experiment.

The capability of UCNP/ICG/RB‐mal to capture TDPAs in vitro was assessed (**Figure** **4**a). A solution of UCNP/ICG/RB‐mal was mixed with 4T1 cell lysates obtained from phototherapy. The hydrate particle size and zeta potential of UCNP/ICG/RB‐mal were measured again 24 h later. The size of the UCNP/ICG/RB‐mal significantly increased and the zeta potential decreased (Figure S7, Supporting Information), indicating TDPAs loading on UCNP/ICG/RB‐mal. In comparison, both the size and zeta potential of UCNP/ICG/RB‐PEG showed little change. Furthermore, quantitative analysis showed that UCNP/ICG/RB‐mal could capture a much higher level of protein (340 µg mg^−1^) than UCNP/ICG/RB‐PEG (Figure S7, Supporting Information). The UCNP/ICG/RB‐mal bond proteins were identified using mass spectrometry and a total of 371 proteins and 21 TDPAs were identified as shown in Figure 4b and Table S1 in the Supporting Information.

Next, the ability of UCNP/ICG/RB‐mal to activate dendritic cells through captured TDPAs was investigated. Bone marrow–derived dendritic cells (BMDCs) from BALB/c mice were collected, and incubated with PBS, UCNP/ICG/RB‐mal@pro, and UCNP/ICG/RB‐PEG@pro for 24 h. Then, the DCs were stained with costimulatory molecules (CD86 and CD40) and analyzed via flow cytometry. As shown in Figure 4c,d, the levels of CD86^+^ and CD40^+^ on the surface of UCNP/ICG/RB‐mal@pro‐treated DCs reached a much higher level than that of UCNP/ICG/RB‐PEG@pro‐treated DCs. Furthermore, tumor necrosis factor α (TNF‐α) secreted by DCs was identified using an enzyme‐linked immune sorbent assay (ELISA). TNF‐α secretion was enhanced by UCNP/ICG/RB‐mal@pro treatment (Figure S8, Supporting Information). Thus, this data confirms that UCNP/ICG/RB‐mal could activate APCs through captured TDPAs.

### 2.4 In Vivo Antitumor Effect of UCNP/ICG/RB‐Mal

**Figure 5:**
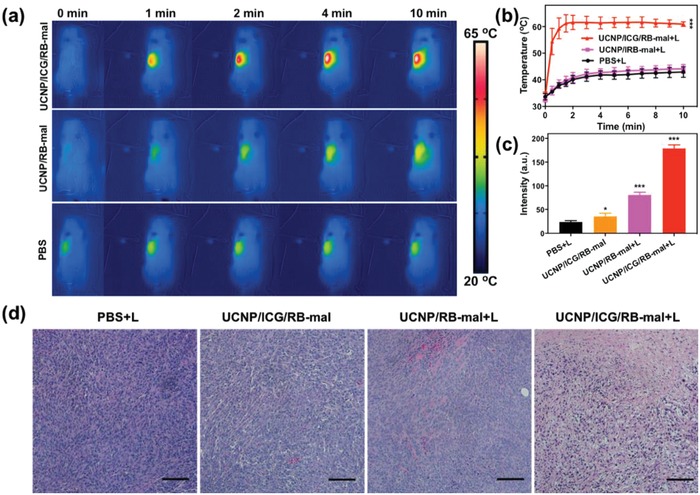
In vivo photothermal and photodynamic response. a) Infrared thermal images of 4T1‐tumor‐bearing mice injected with UCNP/ICG/RB‐mal, UCNP/RB‐mal, or PBS in response to 805 nm laser irradiation (10 min, 0.75W cm^−2^). b) The tumor temperature based on IR thermal imaging data in (a) (***P < 0.001 vs PBS + L). c) The ROS levels of 4T1 tumors injected with UCNP/ICG/RB‐mal, UCNP/RB‐mal, or PBS in response to 805 nm laser irradiation (10 min, 0.75 W cm^−2^; *P < 0.05 vs PBS + L group, ***P < 0.001 vs PBS + L group). d) Representative H&E‐stained images of 4T1 tumors after different treatments as indicated. Scale bar = 200 µm. Data are expressed as mean ± SD (*n* = 4).

An orthotopic breast cancer model of 4T1 in BALB/c mice was used to assess the efficacy of UCNP/ICG/RB‐mal mediated phototherapy. Tumor‐bearing mice were intratumorally injected with either UCNP/RB‐mal or UCNP/ICG/RB‐mal and then irradiated by an 805 nm laser at a power density of 0.75 W cm^−2^ for 10 min. Thermal imaging data showed that the surface temperature of tumors treated via UCNP/ICG/RB‐mal + L increased to 61 °C and remained stable afterward, in comparison to 41 °C when using PBS + L or UCNP/RB‐mal + L (**Figure** **5**a,b). In addition, as shown in Figure 5c, the intertumoral level of ROS was noticeably elevated in mice treated with UCNP/ICG/RB‐mal + L than in mice treated with PBS + L or UCNP/RB‐mal + L. The phototherapy effect was further verified via images of harvested tumor sections one day after treatment. Hematoxylin and eosin (H&E) staining (Figure 5d) showed that tumor structures were severely damaged in the UCNP/ICG/RB‐mal + L group one day after treatment. These results confirm that UCNP/ICG/RB‐mal provided excellent PTT as well as PDT effects.

**Figure 6:**
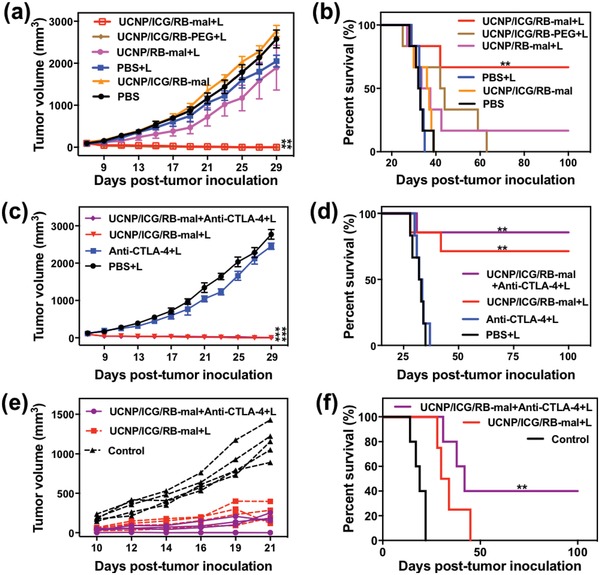
In vivo antitumor effect of UCNP/ICG/RB‐mal. a) Average tumor‐growth curves of different treatment groups of mice with orthotopic 4T1 tumors (*n* = 6, **P < 0.01 vs PBS group). b) Survival curves of different treatment groups of mice bearing orthotopic 4T1 tumors (*n* = 6, **P < 0.01 vs PBS group). c) Average tumor‐growth curves of different groups of treated mice with or without anti‐CTLA‐4 (*n* = 6, ***P < 0.001 vs PBS group). d) Survival curves of different groups of mice‐bearing orthotopic 4T1 tumors in (c) (*n* = 6, **P < 0.01 vs PBS group). e) Growth curves of tumors in individual mice successfully treated mice by phototherapy after tumor cell rechallenge. f) Survival rates of mice in (e). (*n* = 4, **P < 0.01 vs control group.) Data are expressed as mean ± SD.

The effect of UCNP/ICG/RB‐mal was further evaluated by monitoring tumor growth in response to treatment. The tumor growth in mice that received PBS + L or UCNP/ICG/RB‐mal showed no significant difference compared to mice receiving PBS. UCNP/RB‐mal + L slowed tumor growth, as shown in **Figure** **6**a and Figure S9 in the Supporting Information. In contrast, UCNP/ICG/RB‐mal + L completely regressed the tumor without measurable body weight loss (Figure 6a; Figures S9 and S10, Supporting Information). The therapeutic efficacy of both UCNP/RB‐mal + L and UCNP/ICG/RB‐mal + L also translated into improved animal survival of 16% and 67%, respectively (Figure 6b). These findings were consistent with the in vitro phototoxicity evaluation, further verifying that the modification of ICG dramatically improved the anticancer effect of UCNP/ICG/RB‐mal as a mediator of phototherapy. Both UCNP/ICG/RB‐mal and UCNP/ICG/RB‐PEG + L had the identical effect on treated primary tumors. However, only UCNP/ICG/RB‐mal + L yielded long‐term survival (Figure 6b), while all mice in the UCNP/ICG/RB‐PEG + L group died within 60 days, due to lung metastasis (Figure S11, Supporting Information). This result demonstrates that ‐mal modification could inhibit tumor metastasis.

To further enhance the ability of UCNP/ICG/RB‐mal, checkpoint inhibitor anti‐CTLA‐4 was combined with UCNP/ICG/RB‐mal mediated phototherapy for the treatment of 4T1 mouse tumors. As shown in Figure 6d, the use of anti‐CTLA‐4 further enhanced the therapeutic effect of UCNP/ICG/RB‐mal + L, thus increasing the survival rate from 67% to 83%. More importantly, two mice in the UCNP/ICG/RB‐mal + L + anti‐CTLA‐4 group successfully rejected tumor rechallenge (via subcutaneous injection of 2 × 10^5^ 4T1 cells), demonstrating that UCNP/ICG/RB‐mal + anti‐CTLA‐4 + L can induce durable antitumor immunity (Figure 6f).

**Figure 7:**
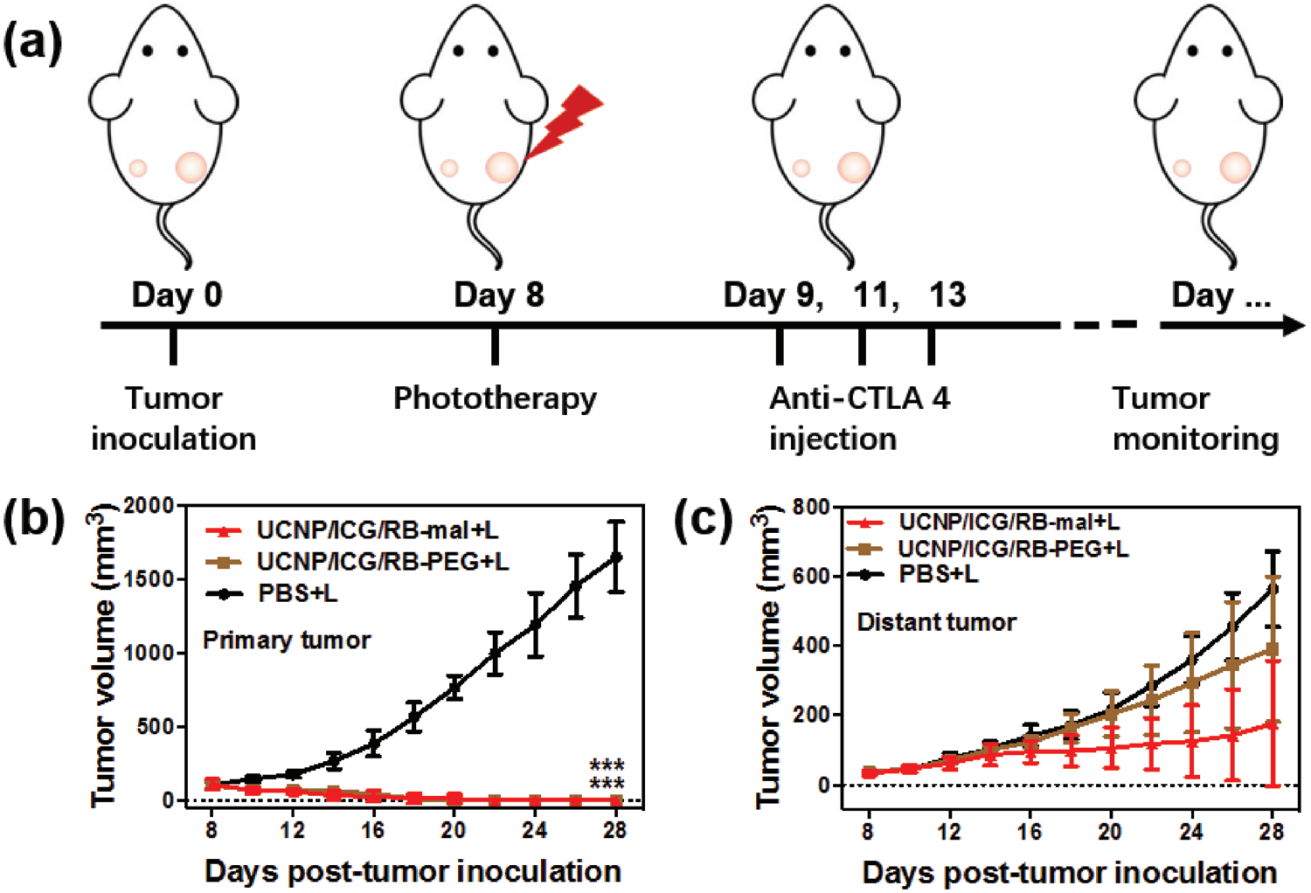
Abscopal effect of UCNP/ICG/RB‐mal based phototherapy in combination with checkpoint inhibition for simultaneously inoculated tumors. a) Schematic depiction of the experimental approach for the evaluation of the abscopal effect induced by UCNP/ICG/RB‐mal based phototherapy. b) Growth curves of primary tumors of mice after various treatments. (***P < 0.001 vs PBS + L group.) c) Growth curves of distant tumors on mice in different treated groups. Data are expressed as mean ± SD (*n* = 5).

Furthermore, the abscopal effect of UCNP/ICG/RB‐mal based phototherapy was explored (**Figure** **7**a). A bilateral 4T1 tumor model was developed via subcutaneous injection of 4T1 cells into both the left and right flank regions of BALB/c mice. The right tumor was defined as the primary tumor and the left tumor was defined as the distant tumor. On day 8, the primary tumors were treated with PBS + L, UCNP/ICG/RB‐PEG + L, and UCNP/ICG/RB‐mal + L. Subsequently, mice received intraperitoneal injection with anti‐CTLA‐4 at doses of 30 mg per mouse three times on days 9, 11, and 13. UCNP/ICG/RB‐mal + L treatment significantly inhibited the growth of primary tumors (Figure 7b; Figure S12, Supporting Information) and slowed the growth of distant tumors (Figure 7c; Figure S12, Supporting Information). In summary, these results indicate that UCNP/ICG/RB‐mal based phototherapy can effectively eradicate tumors and inhibit tumor metastasis by inducing antitumor immunity.

### 2.5 Antitumor Immune Responses Induced by UCNP/ICG/RB‐Mal

**Figure 8:**
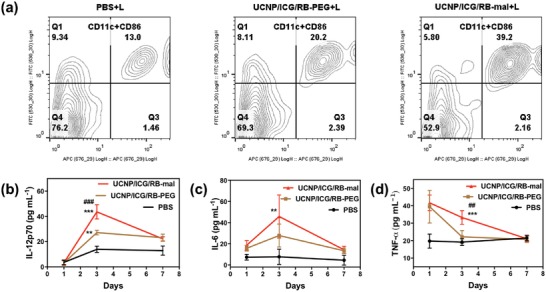
Immune responses after UCNP/ICG/RB‐mal based phototherapy. a) DC maturation induced by UCNP/ICG/RB‐mal based phototherapy treatment of mice‐bearing 4T1 tumors. Cells in the tumor were collected 24 h after various treatments for assessment via flow cytometry after staining with CD11c and CD86. b–d) Cytokine levels in sera from mice isolated at 1, 3, and 7 days post different treatments, respectively (**P < 0.01 vs PBS group, ***P < 0.001 vs PBS group, ^##^ *P* < 0.01 vs UCNP/ICG/RB‐PEG group, ^###^ *P* < 0.001 vs UCNP/ICG/RB‐PEG group, test at 3 days). Data are expressed as mean ± SD (*n* = 4).

The UCNP/ICG/RB‐mal induced immune responses were investigated via flow cytometry, ELISA, and immunofluorescence staining assay. DC maturation was studied via flow cytometry. As shown in **Figure** **8**a, UCNP/ICG/RB‐mal + L significantly increased the level of DC maturation, and levels were 1.55‐fold and 3.01‐fold higher than those of the UCNP/ICG/RB‐PEG + L group and the PBS+L group, respectively. The increase of infiltration level of DCs by UCNP/ICG/RB‐mal + L was demonstrated by the CD11c^+^ expression in treated tumors (Figure S14, Supporting Information). The antitumor immunity evoked by UCNP/ICG/RB‐based phototherapy was further studied by measuring the serum concentration of cytokines via ELISA. Compared to PBS + L control, UCNP/ICG/RB‐based phototherapy promoted the sustained secretion of tumor necrosis factor α, interleukin 6 (IL‐6), and interleukin 12 (IL‐12) (Figure 8b–d). As compared with UCNP/ICG/RB‐PEG + L group, their cytokines induced by UCNP/ICG/RB‐mal based phototherapy were obviously higher, suggesting that UCNP/ICG/RB‐mal based phototherapy is favorable for triggering antitumor immune response.

**Figure 9:**
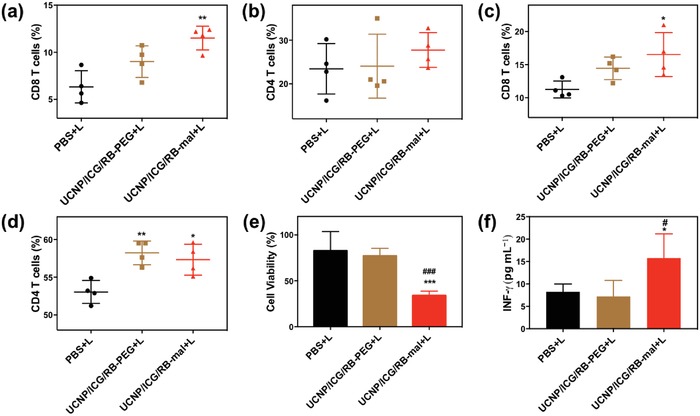
Antitumor immunity of UCNP/ICG/RB‐mal based phototherapy. a,b) Flow cytometric analysis of the relative abundance of CD8^+^ and CD4^+^ T‐cell subpopulations in untreated secondary tumors. T cells were defined as being CD45^+^CD3^+^ (**P < 0.01 vs PBS + L group). c,d) Flow cytometric analysis of relative abundance of CD8^+^ and CD4^+^ T‐cell subpopulations in spleens. T cells were defined as being CD3^+^ (*P < 0.05 vs PBS + L group, **P < 0.01 vs PBS + L group). e) 4T1 tumor cells viability after incubation for 24 h with splenocytes collected from mice after different treatments (***P < 0.001 vs PBS + L group, ^###^ *P* < 0.001 vs UCNP/ICG/RB‐PEG + L group). f) INF‐γ levels secreting by CD4^+^ and CD8^+^ T cells in splenocytes after being incubated with 4T1 cells (*P < 0.05 vs PBS + L group, ^#^ *P* < 0.05 vs UCNP/ICG/RB‐PEG + L group). Data are expressed as mean ± SD (*n* = 4).

T cell activation was further investigated after UCNP/ICG/RB‐mal based phototherapy. In particular, the relative abundance of activated T cells was measured in untreated secondary tumors and spleens of treated mice seven days after various treatments of the primary tumors. In the secondary tumors, more infiltrating CD8^+^ T cells were found in the UCNP/ICG/RB‐mal + L treatment group compared to other treatment groups (**Figure** **9**a). Immunofluorescence staining showed a significantly higher expression of CD8^+^ T cells in the secondary tumors in the UCNP/ICG/RB‐mal + L group. In addition, the percentage of regulatory T cells (T_reg_) in the secondary tumors showed a significant decrease for the UCNP/ICG/RB‐mal + L treatment group compared to that of the other groups (Figure S17a, Supporting Information), and the CD8^+^/T_reg_ ratio was significantly increased for the UCNP/ICG/RB‐mal + L treatment group compared to other groups (Figure S17b, Supporting Information). Additionally, more infiltrating CD8^+^ T cells in the UCNP/ICG/RB‐mal + L treatment group were found in the spleens (Figure 9c). These results indicate that UCNP/ICG/RB‐mal based phototherapy effectively induced differentiation of T cells to CD8^+^ T cells and significantly improved the intratumoral CD8^+^ T cell ratios.

Furthermore, the systemic effect of UCNP/ICG/RB‐mal based phototherapy was assessed. Splenocytes isolated from mice after different treatments were incubated with 4T1 cells. After incubation for 24 h, more than 75% of 4T1 cells were killed by the splenocytes from mice in the UCNP/ICG/RB‐mal + L group (Figure 9e). Furthermore, a significantly higher level of INF‐γ was observed secreted by splenocytes from mice in the UCNP/ICG/RB‐mal + L groups (Figure 9f). These findings imply that UCNP/ICG/RB‐mal based phototherapy can elicit systemic T‐cell activation.

## 3 Conclusion

In this study, UCNP/ICG/RB‐mal, used in combination with an NIR light irradiation, effectively eliminated primary tumors and induced antitumor immunity. UCNPs as NIR‐triggered mediators converted NIR light to visible light to activate photosensitizer molecules. ICG is a well‐studied fluorescent dye that enhances the UCL of UCNPs and achieves selective PTT. RB is a photosensitizer molecule, which has been widely utilized for PDT of various tumors. DSPE‐PEG‐mal is used to capture tumor‐derived protein antigens by forming stable thioether bonds. Upon NIR laser irradiation, UCNP/ICG/RB‐mal can efficiently generate ^1^O_2_ and induce a significant temperature increase, thus killing tumor cells by combined PDT and PTT effects. Tumor‐derived protein antigens were successfully captured by UCNP/ICG/RB‐mal nanoparticles and promoted cancer immunity. Administration of the UCNP/ICG/RB‐mal through intratumoral injection and followed by NIR laser‐triggered activation demonstrated efficient destruction of primary tumors and inhibition of metastasis by simultaneously boosting specific immune responses and blocking CTLA‐4‐dependent immune evasion. Furthermore, UCNP/ICG/RB‐mal based phototherapy strategy shows a strong long‐term immune memory function, which protects the treated mice against tumor cell rechallenge. Hence, antigen‐capturing UCNP‐based phototherapy strategy could effectively kill primary tumors in response to NIR light exposure, and elicit effective antimetastatic effects by stimulating systemic antitumor immunity. The NIR‐triggered antigen‐capturing nanoplatform proposed in this study might offer a new strategy for the improvement of the therapeutic outcomes and the inhibition of metastasis tumor recurrence.

## 4 Experimental Section

*Materials*: Y(CH_3_CO_2_)_3_⋅*x*H_2_O (99.9%), Yb(CH_3_CO_2_)_3_⋅*x*H_2_O (99.9%), Er(CH_3_CO_2_)_3_⋅*x*H_2_O (99.9%), Nd(CH_3_CO_2_)_3_⋅*x*H_2_O (99.9%), NaOH (98%), NH_4_F (98%), ICG, rose bengal, 1‐octadecene (90%), oleic acid (90%), dichloromethane (CH_2_Cl_2_), and ethanol were purchased from Sigma‐Aldrich (USA). 1,2‐distearoyl‐sn‐glycero‐3‐phosphoethanolamine‐*N*‐[amino(polyethylene glycol)2000] (DSPE‐PEG), and 1,2‐distearoyl‐sn‐glycero‐3‐phosphoethanolamine‐*N*‐[maleimide(polyethylene glycol)‐2000] (DSPE‐PEG‐mal) were purchased from Avanti Polar Lipids (USA). Unless otherwise mentioned, all chemicals were used as received without further purification.

*Cell Line and Animals*: The 4T1 cell line was obtained from the American Type Culture Collection (Rockville, USA), where this line was authenticated via morphology, karyotyping, and PCR‐based approaches and tested for mycoplasma. 4T1 cells were cultured in RPMI 1640 (Gibco, USA) supplemented with 10% fetal bovine serum (FBS), 100 U mL^−1^ penicillin, and 100 µg mL^−1^ streptomycin (Gibco, USA). Cell cultures were maintained below a 50% confluence and early passage cultures (between four and nine) were utilized for the experiments. BALB/c female mice (6 weeks, 18–20 g) were provided from Harlan Sprague Dawley Co. (USA). Mice were housed in the animal facility of the Department of Comparative Medicine at the University of Oklahoma Health Sciences Center (OUHSC, Oklahoma, USA). All experiments were conducted in compliance with the Guide for the Care and Use of Laboratory Animals published by the NIH and approved by the OUHSC Institutional Animal Care and Use Committee.

*Synthesis of NaYF_4_:48%Yb/2%Er Core Nanoparticles*: In a typical procedure for the synthesis of NaYF_4_:48%Yb/2%Er nanoparticles, 2 mmol Ln(CH_3_CO_2_)_3_ (Ln = Y, Yb, and Er) was added to a 100 mL flask, containing 12 mL oleic acid. The mixture was heated at 150 °C for 30 min to remove the water from the solution. A solution of 1‐octadecene (15 mL) was then quickly added to the flask and the resulting mixture was heated at 150 °C for a further 30 min before cooling down to 50 °C. Shortly thereafter, 15 mL of methanol solution containing both NH_4_F (9 mmol) and NaOH (5 mmol) was added and the resultant solution was stirred for 30 min. After methanol had evaporated, the solution was heated to 300 °C under argon atmosphere for 1.5 h and then cooled to room temperature. The resulting nanoparticles were precipitated via ethanol addition, collected via centrifugation at 6000 rpm for 5 min, washed with ethanol three times, and redispersed in 20 mL of cyclohexane.

*Synthesis of NaYF_4_:48%Yb/2%Er@NaYF_4_:30%Nd Core/Shell Nanoparticles*: The NaYF_4_:30%Nd shell precursor was first prepared by mixing 1 mmol Ln(CH_3_CO_2_)_3_ (Ln = Y, Nd) and 12 mL oleic acid in a 100 mL flask followed by heating at 150 °C for 30 min. Then, 1‐octadecene (15 mL) was added and the mixed solution was heated at 150 °C for further 30 min before cooling to 50 °C. Subsequently, NaYF_4_:Yb/Er core nanoparticles (1 mmol) dispersed in 10 mL of cyclohexane were added along with a 10 mL methanol solution of NH_4_F (4.5 mmol) and NaOH (2.5 mmol). The resulting mixture was stirred at 50 °C for 30 min, at which time, the solution was heated to 300 °C under argon atmosphere for 1.5 h and then cooled to room temperature. The resulting nanoparticles were precipitated via addition of ethanol, collected by centrifugation at 6000 rpm for 5 min, washed with ethanol three times, and redispersed in 20 mL of cyclohexane.

*Synthesis of UCNP/ICG/RB‐Mal Nanoparticles*: NaYF_4_:48%Yb/2%Er@NaYF_4_:30%Nd core/shell nanoparticles were dispersed in DCM (5 mL, 4 mg mL^−1^), followed by mixing with DSPE‐PEG2K, DSPE‐PEG2K‐mal, and ICG at concentrations at 4, 4, and 0.4 mg mL^−1^, respectively. Subsequently, the resulting mixture was sonicated for about 5 min, and the solvent of DCM was fully removed via rotary evaporator. The residual solid was hydrated by adding 4 mL purified water and sonicated for 30 min. The resulting nanoparticles (UCNP/ICG‐mal) were collected by centrifugation 13 000 rpm for 10 min, washed with purified water three times, and redispersed in 4 mL PBS. For RB loading, 4 mg RB was added to UCNP/ICG‐mal solution (2 mL, 5 mg mL^−1^ in PBS) and stirred at room temperature overnight. Then, the UCNP/ICG/RB‐mal nanoparticles were collected via centrifugation at 13 000 rpm for 10 min, washed with purified water three times, and redispersed in PBS.

*Characterization of Nanoparticles*: Powder X‐ray diffraction patterns of samples were collected with an X‐ray diffractometer (MiniFlex2, Rigaku, Japan) with Cu Kα1 radiation (λ = 0.154187 nm). Both the low‐ and high‐resolution TEM measurements were conducted via JEOL‐2010 TEM (JEOL, Japan). The amounts of loaded ICG and RB were quantitatively evaluated via UV–vis–NIR spectrophotometer (Cary 50 Bio, USA). The loading capacity of ICG (RB) was calculated using the following equation: loading capacity of ICG (RB) (%) = (weight of loaded ICG (RB))/(weight of UCNP/ICG/RB‐mal) × 100%. UCL spectra were measured by FLS980 spectrometer upon 805 nm excitation provided by a CW semiconductor laser.

*Detection of Singlet Oxygen*: DPBF were used to react with ^1^O_2_ to detect ROS in the aqueous solution. The UCNP‐mal, UCNP/RB‐mal, UCNP/ICG‐mal, and UCNP/ICG/RB‐mal nanoparticles (500 µg) in 1 mL water containing 0.1 mmol of DPBF dye were used for a typical DPBF experiment.^41^ The solution was placed in a cuvette, stirred, and irradiated with a 1 W cm^−2^ using an 805 nm laser (AngioDynamics, USA). ROS production was measured through the absorption of DPBF at 420 nm by UV–vis–NIR spectrophotometer (Cary50 Bio, USA). For intracellular ROS detection, DCFH‐DA was used as ROS sensor. 4T1 cells were seeded into 8‐well chambered slides (Thermo Scientific, USA) and cultured for 12 h. After removal of the medium, cells were treated with PBS, UCNP/RB‐mal, UCNP/ICG‐mal, or UCNP/ICG/RB‐mal for 4 h and then irradiated with an 805 nm laser for 3 min (at 0.75 W cm^−2^). The cells were further incubated with DCFH‐DA (20 × 10^−6^ m) for 30 min and analyzed via fluorescence microscopy (Olympus, Japan).

*In Vitro Phototoxicity of UCNPs/ICG/RB‐Mal Nanoparticles*: The phototoxicities of UCNP/RB‐mal, UCNP/ICG‐mal, and UCNP/ICG/RB‐mal nanoparticles upon laser irradiation were evaluated against 4T1 cells. Nanoparticles at doses varying from 0 to 200 µg mL^−1^ were incubated with cells for 4 h before the cell culture medium was replaced with fresh medium. The cells were submitted to 805 nm laser irradiation at 0.75 W cm^−2^ for 5 min and then incubated for 24 h before their viability was determined with a CCK‐8 assay.

*Detection of ICD Biomarkers*: Surface‐exposure of CRT was assessed via immunofluorescence, where 4T1 cells were seeded into 8‐well chambered slides and cultured for 12 h. The cells were treated with PBS, UCNP/RB‐mal, UCNP/ICG‐mal, or UCNP/ICG/RB‐mal for 4 h of incubation and irradiated with an 805 nm laser for 5 min (at 0.75 W cm^−2^). Cells treated with UCNP/ICG/RB‐mal without laser irradiation served as dark control. After a further 24 h of incubation, cells were washed twice with PBS and then incubated with anticalreticulin antibody for 2 h at 4 °C. Subsequently, the cells were washed twice with PBS and incubated with Alexa Fluor 488‐conjugated secondary antibody (BioLegend, USA) for 1 h. After staining with DAPI, the cells were analyzed via fluorescence microscopy (Olympus, Japan). Extracellular HMGB1 in conditioned media (serum‐free) secreted from treated cells were measured via HMGB1 ELISA Kit (R&D systems), following the manufacturer's instructions.

*In Vitro Formulation of UCNPs Coated with Tumor‐Derived Protein Antigens*:^28^ 4T1 cells were incubated with RB and ICG in six well plates for 4 h; then, the 4T1 cells were washed with PBS and irradiated with 540 nm LED light (200 J cm^−2^) and an 805 nm laser (0.75 W cm^−2^) for 5 min. Subsequently, the 4T1 cells were incubated in RPMI 1640 media without FBS for 48 h. After incubation, the supernatant was collected and spun down at 200 g for 5 min to remove all insoluble cellular debris. UCNPs were incubated with antigen‐containing supernatants. Specifically, 20 mg of each of the UCNP formulations was mixed with 12 mL of the tumor antigens from 12 × 10^6^ irradiated 4T1 cells. After incubation, UCNPs were washed with PBS three times, and redispersed in PBS.

*Characterization of UCNP/ICG/RB‐Mal before and after Ex Vivo Antigen Capture*: Zeta potential and hydrodynamic diameter distribution of the UCNPs/ICG/RB‐mal after antigen capture were determined via dynamic light scattering measurement (Nano ZS ZEN3600, Malvern, UK). The amount of protein bound by the UCNPs/ICG/RB‐mal was measured using the Bradford method with bovine serum albumin as standard. Specifically, the total protein uptake by the UCNP/ICG/RB‐mal@pro was determined by subtracting the protein concentration in the supernatant after UCNP/ICG/RB‐mal capture from the protein concentration in the supernatant before capture. All presented values are based on the average of three separate measurements. The proteins bound by the UCNPs were identified via liquid chromatography/tandem mass spectroscopy (LC/MS/MS) on an AB SCIEX nano LC‐MS/MS (Triple TOF 5600 plus) system (SCIEX, USA) and the original MS/MS file data were analyzed via ProteinPilot Software v4.5.

*In Vitro Dendritic Cell Stimulation*: Bone marrow–derived dendritic cells were generated from 8 week old BALB/c female mice, according to an established method.^42^ For in vitro DC stimulation experiments, UCNP/ICG/RB‐PEG@pro or UCNP/ICG/RB‐mal@pro at a dose of 250 µg mL^−1^ were incubated with 10^6^ BMDCs for 24 h. After various treatments, BMDCs stained with anti‐CD11c‐FITC, anti‐CD86‐PE, anti‐CD11c‐FITC, or anti‐CD40‐Pacblue antibody were analyzed via Stratedigm S1200Ex flow cytometer (Stratedigm, USA).

*Phototherapy Study in a 4T1 Orthotopic Murine Breast Cancer Model*: BALB/c mice were subcutaneously injected with 5 × 10^5^ 4T1 cells into the right breast pad. When tumors reached 100–150 cm^3^, mice were randomly divided into three groups (*n* = 4) and UCNP/RB‐mal, UCNP/ICG/RB‐mal, or PBS was intratumorally injected into mice. Two hours after injection, mice were anaesthetized with 2% v/v isoflurane and primary tumors were irradiated with an 805 nm laser for 10 min (at 0.75 W cm^−2^). The temperature on the tumor surface during PTT was measured with an infrared thermal camera E80 (FLIR, Boston, USA). After irradiation, tumors were immediately collected and homogenized in cold PBS. Subsequently, the homogenate was centrifuged at 3000 rpm for 10 min and the supernatant was harvested to determine ROS using assay kits according to the manufacturer's instructions.

*Anticancer Efficacy in the 4T1 Orthotopic Murine Breast Cancer Model*: BALB/c mice were subcutaneously injected with 5 × 10^5^ 4T1 cells into the right breast pad. When tumors reached 100–150 cm^3^, mice were randomly assigned to their respective group and UCNP/RB‐mal, UCNP/ICG/RB‐PEG, UCNP/ICG/RB‐mal, or PBS was intratumorally injected into the mice. Two hours after injection, mice were anaesthetized with 2% v/v isoflurane and primary tumors were irradiated with an 805 nm laser for 10 min (at 0.75 W cm^−2^). Mice in UCNP/ICG/RB‐mal groups were intraperitoneally injected with anti‐CTLA‐4 (30 mg per mouse) one, three, and five days after treatment. The sizes of tumors were measured every two days using a digital caliper, and the tumor volume was estimated via ellipsoidal calculation as *V* = (width)^2^ × length × π/6. The mice were euthanized when the tumors reached the maximum permitted size (2.0 cm in any dimension) or when ulcerations occurred.

*Tumor Rechallenge*: The surviving mice were rechallenged with 2 × 10^5^ 4T1 cells on the right flank 100 days after the first tumor inoculation to examine immunological memory effects. The tumor sizes were measured every two days. The mice were euthanized when the tumors reached the maximum permitted size (2.0 cm in any dimension) or when ulcerations occurred.

*Abscopal Effect of UCNP/ICG/RB‐Mal*: BALB/c mice were subcutaneously injected with 5 × 10^5^ 4T1 cells into the right flank (primary tumor) and injected with 1 × 10^5^ 4T1 cells into the left flank (distant tumor). After primary tumor inoculation for eight days, bilateral 4T1 tumor bearing mice (*n* = 5) were intratumorally injected with UCNP/ICG/RB‐PEG, UCNP/ICG/RB‐mal, or PBS. Mice were anaesthetized with 2% v/v isoflurane and primary tumors were irradiated with an 805 nm laser for 10 min (0.75 W cm^−2^) 2 h after injection. Mice were intraperitoneally injected with anti‐CTLA‐4 (30 mg per mouse) one, three, and five days after treatment. The primary and distant tumor sizes and body weights were recorded every two days.

*Antitumor Immunity Study in a Bilateral 4T1 Tumor Model*: BALB/c mice were subcutaneously injected with 5 × 10^5^ 4T1 cells into the right flank (primary tumor) and left (contralateral tumor) flank, respectively. When the primary tumors reached 100–150 mm^3^, mice were randomly assigned to their respective group (*n* = 4) and UCNP/ICG/RB‐PEG, UCNP/ICG/RB‐mal, or PBS was intratumorally injected into the mice. Mice were anaesthetized with 2% v/v isoflurane and primary tumors were irradiated with an 805 nm laser for 10 min (at 0.75 W cm^−2^) 2 h after injection. Mice were intraperitoneally injected with anti‐CTLA‐4 at a dose of 30 mg per mouse one day after treatment. Tumors, spleen, and blood were collected on days 1, 3, and 7 following laser irradiation for the analysis of DC cells, T cells, and immune serum, respectively.

*Flow Cytometry*: Tumors were harvested, treated with 1 mg mL^−1^ collagenase I (Gibco, USA) for 30 min, and grounded using the rubber end of a syringe. Spleens were also harvested and grounded, and red blood cells were removed via ACK lysis buffer. Cells were filtered through nylon mesh filters and washed with PBS. The single‐cell suspension was incubated with anti‐CD16/32 (clone 93; eBioscience, USA) to reduce nonspecific binding to Fc receptors (FcRs). To analyze activated DCs, the cells were stained with antimouse CD11c‐APC and antimouse CD86‐FITC antibodies. To analyze the active T cells in tumors, the cells were stained with Live/Dead‐BV510, antimouse CD45‐BV421, antimouse CD3‐AF700, antimouse CD4‐APC, and antimouse CD8a‐FITC. For analysis of active T cells in spleens, the cells were stained with Live/Dead‐BV510, antimouse CD3‐AF700, antimouse CD4‐APC, and antimouse CD8a‐FITC. A Stratedigm S1200Ex flow cytometer (Stratedigm, USA) was used for flow cytometry, and data analysis was conducted using FlowJo software.

*Cytokine Detection*: Serum samples were isolated from mice after various treatments and were diluted prior to analysis. Tumor necrosis factor α, interleukin 6, interleukin 12, and interferon gamma were analyzed via ELISA kits (R&D Systems, USA) according to the manufacturer's protocol.

*Immunofluorescence Assay*: Tumors were collected and frozen tissue sections of 6 mm thickness were prepared via cryostat. These sections were air‐dried for at least 1 h and then fixed in acetone for 10 min at room temperature. After blocking with 20% donkey serum, the sections were incubated with antibodies, washed twice with PBS, and observed via fluorescence microscopy (Olympus, Japan).

*Statistical Analysis*: Values are expressed as mean ± standard error of the mean. The data was analyzed using GraphPad Prism software (GraphPad Software Inc., La Jolla, CA, USA). One‐way analysis of variance (ANOVA) with Tukey's post hoc pairwise comparisons was employed to compare the groups. A *P* value of <0.05 was considered statistically significant.

## Conflict of Interest

The authors declare no conflict of interest.

## Supporting information

Supplementary
