# Supplementary material for: NIR‐Triggered Phototherapy and Immunotherapy via an Antigen‐Capturing Nanoplatform for Metastatic Cancer Treatment
Source: Adv Sci (Weinh). 2019 Mar 13;6(10):1802157. doi: 10.1002/advs.201802157 (PMC6523374; doi:10.1002/advs.201802157)
Supplement: Supplementary file 1 — Supplementary [file ADVS-6-1802157-s001.pdf]

Copyright WILEY-VCH Verlag GmbH & Co. KGaA, 69469 Weinheim, Germany, 2019.

ADVANCED SCIENCE
Open Access

Supporting Information

for *Adv. Sci.,* DOI: 10.1002/advs.201802157

NIR-Triggered Phototherapy and Immunotherapy via an Antigen-Capturing Nanoplatform for Metastatic Cancer Treatment

Meng Wang, Jun Song, Feifan Zhou, Ashley R. Hoover, Cynthia Murray, Benqing Zhou, Lu Wang, Junle Qu,* and Wei R. Chen**

Copyright WILEY-VCH Verlag GmbH & Co. KGaA, 69469 Weinheim, Germany, 2019.

Supporting Information

NIR-triggered phototherapy and immunotherapy via antigen-capturing nanoplatform for metastatic cancer treatment

Meng Wang, Jun Song,* Feifan Zhou, Ashley Hoover, Cynthia Murray, Benqing Zhou, Lu Wang, Junle Qu,* and Wei R. Chen*

Supplementary Figures

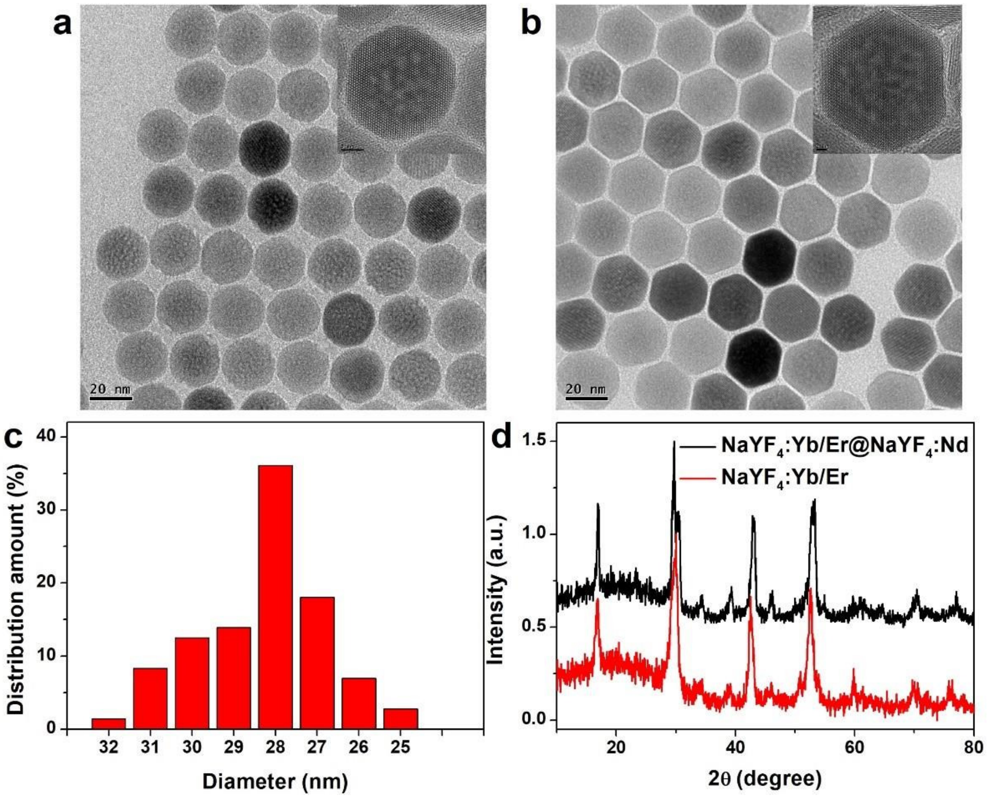

Figure S1. Transmission electron microscope (TEM) and High Resolution Transmission Electron Microscope (HRTEM) images of a) oleate-capped $NaYF_4$:Yb/Er UCNPs b) oleate-capped $NaYF_4$:Yb/Er@$NaYF_4$:Nd UCNPs. c) Size distribution of oleate-capped $NaYF_4$:Yb,Er/$NaYF_4$:Nd UCNPs as determined from 200 particles in the TEM image. d) Powder XRD pattern of $NaYF_4$:Yb/Er UCNPs and $NaYF_4$:Yb/Er@$NaYF_4$:Nd UCNPs.

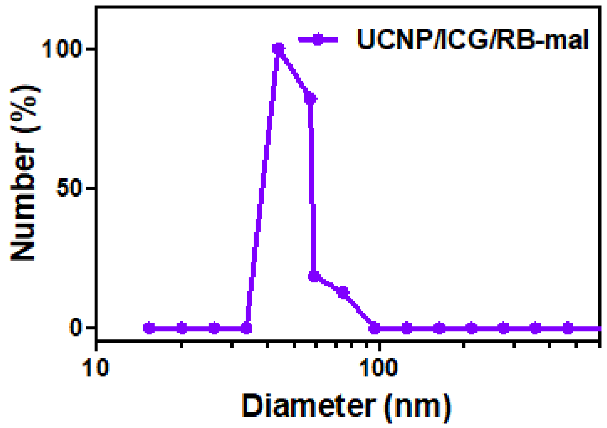

Figure S2. Hydrodynamic diameter distribution of UCNP/ICG/RB-mal. The hydrodynamic diameters of UCNP/ICG/RB-mal were determined to be 41 ± 1 nm.

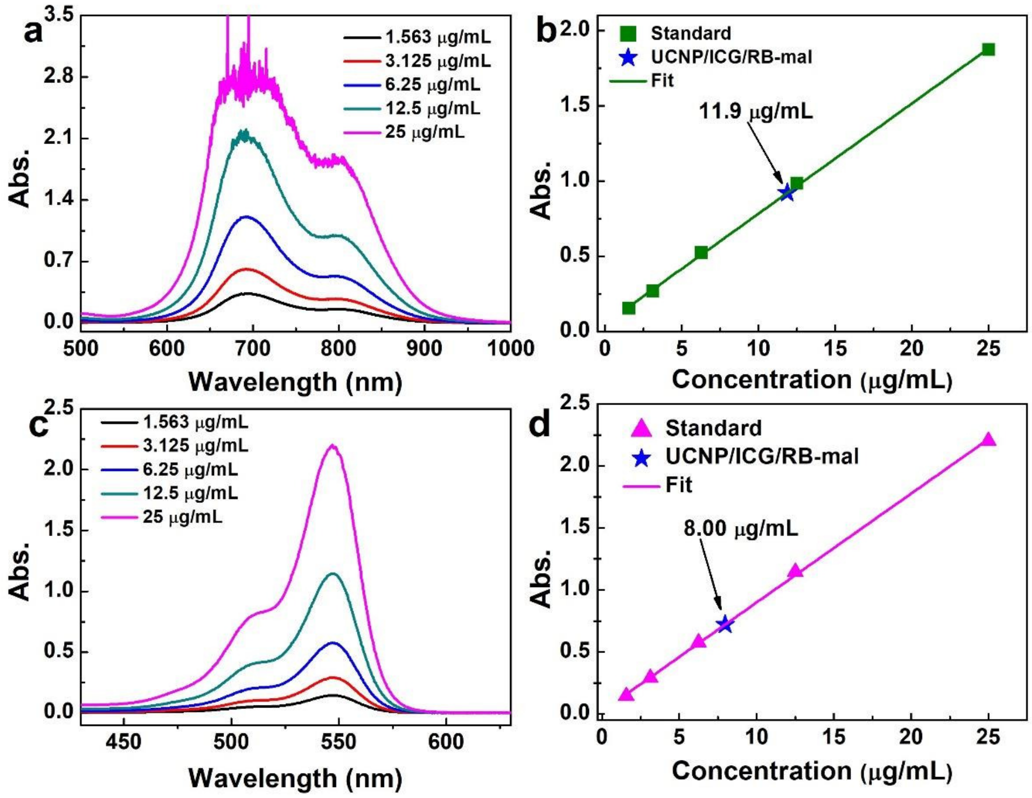

Figure S3. Quantitative analysis of indocyanine green (ICG) or rose bengal (RB) loaded in UCNP/ICG/RB-mal. a) Absorption spectra of ICG solution. b) The standard curve of absorption for ICG. The loading amount of ICG was determined to be 1.19% (w / w) in UCNP/ICG/RB-mal. c) Absorption spectra of RB solution. d) The standard curve of absorption for RB. The loading amount of RB was determined to be 0.8% (w / w) in UCNP/ICG/RB-mal.

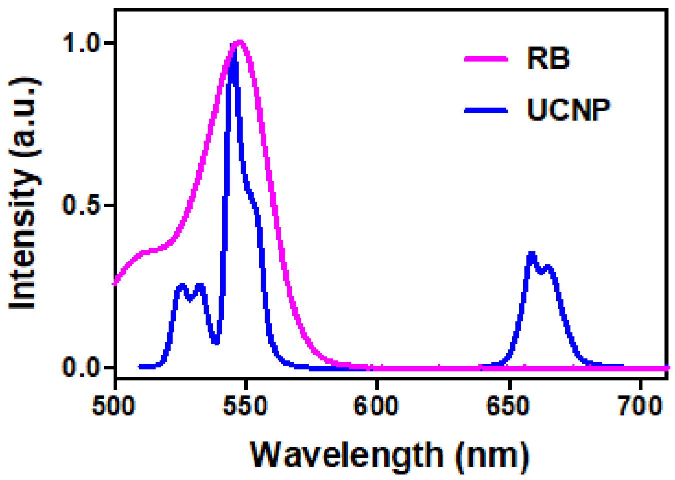

Figure S4. Absorption spectra of the RB solution and upconversion luminescence (UCL) spectra of oleate-capped $NaYF_4$:Yb/Er@$NaYF_4$:Nd nanoparticles, respectively.

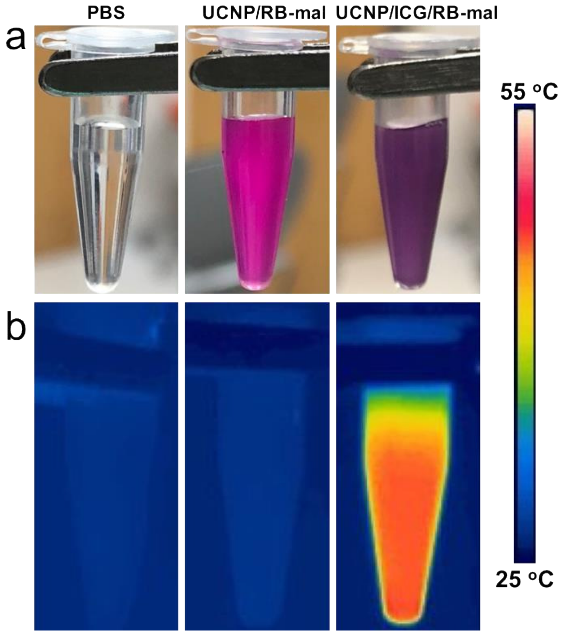

Figure S5. Infrared thermal images of PBS, UCNP/RB-mal, and UCNP/ICG/RB-mal solutions upon a 805-nm laser irradiation for 4 min. (0.75 W cm^{-2}).

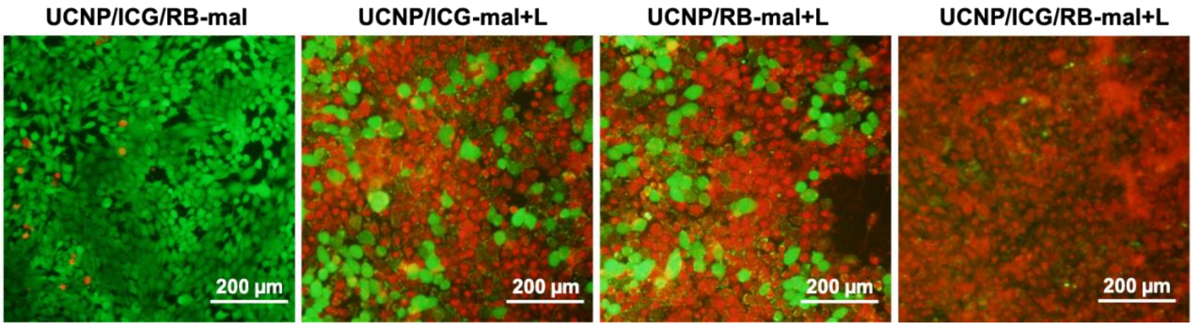

Figure S6. Fluorescence images of 4T1 cells after 4 h incubation with UCNP/ICG-mal, UCNP/RB-mal, and UCNP/ICG/RB-mal exposed to a 805-nm laser for 5 min. (0.75 W cm^{-2}) The cells were con-stained by calcein AM (green, live cells) and propidium iodide (red, dead cells) before imaging. Scale bar: 200 μm.

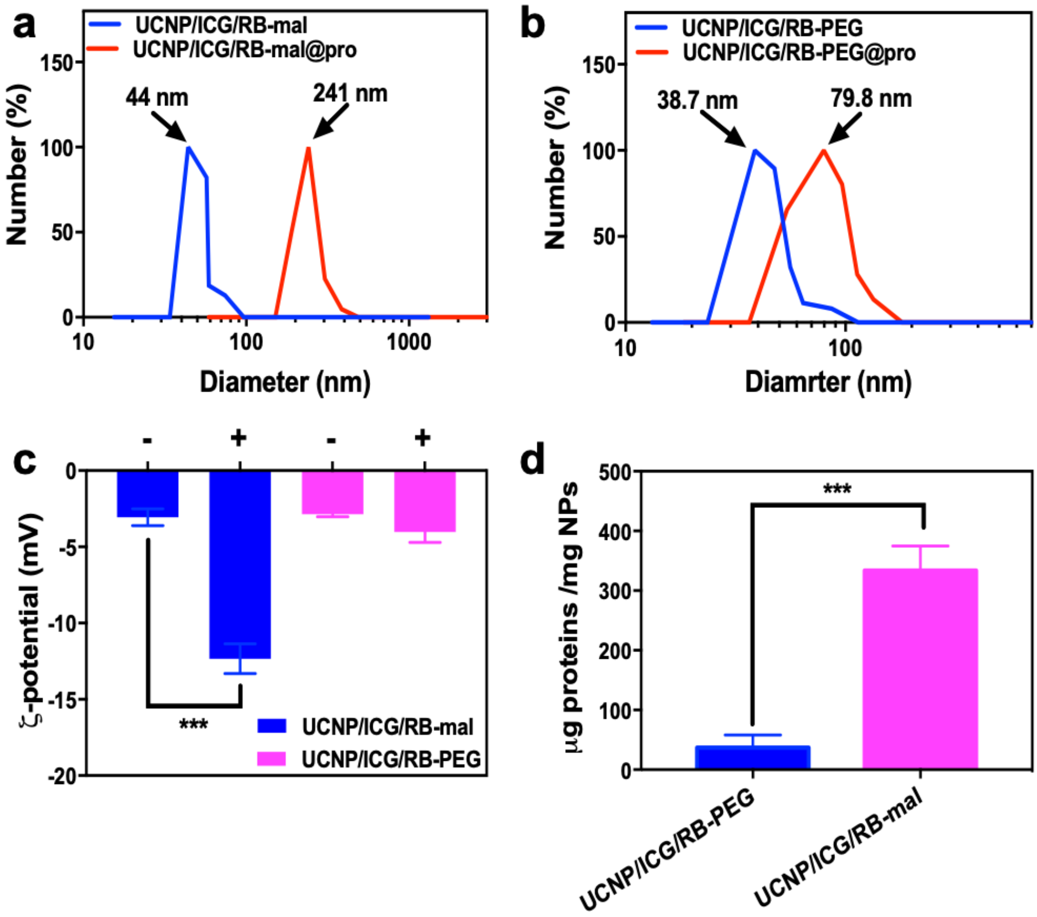

Figure S7. The size of a) UCNP/ICG/RB-mal and b) UCNP/ICG/RB-PEG before and after antigen capture. c) The zeta potential of UCNP/ICG/RB-mal or UCNP/ICG/RB-PEG before and after antigen capture. (***P < 0.001) d) Quantification of protein captured by nanoparticles. (***P < 0.001). Data are expressed as means ± SD (n = 4).

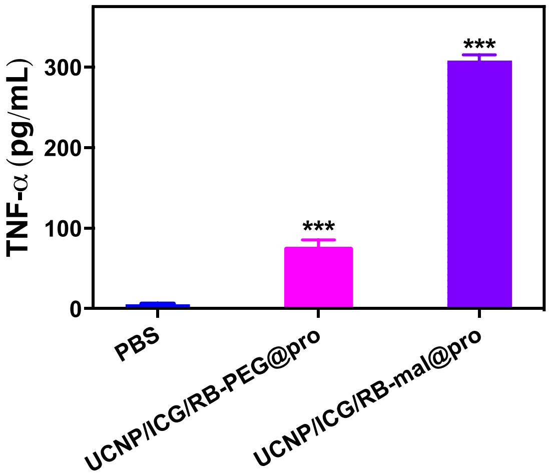

Figure S8. Secretion of TNF-α in BMDC suspensions measured by ELISA (***P < 0.001 vs PBS). Data are expressed as means ± SD (n = 4).

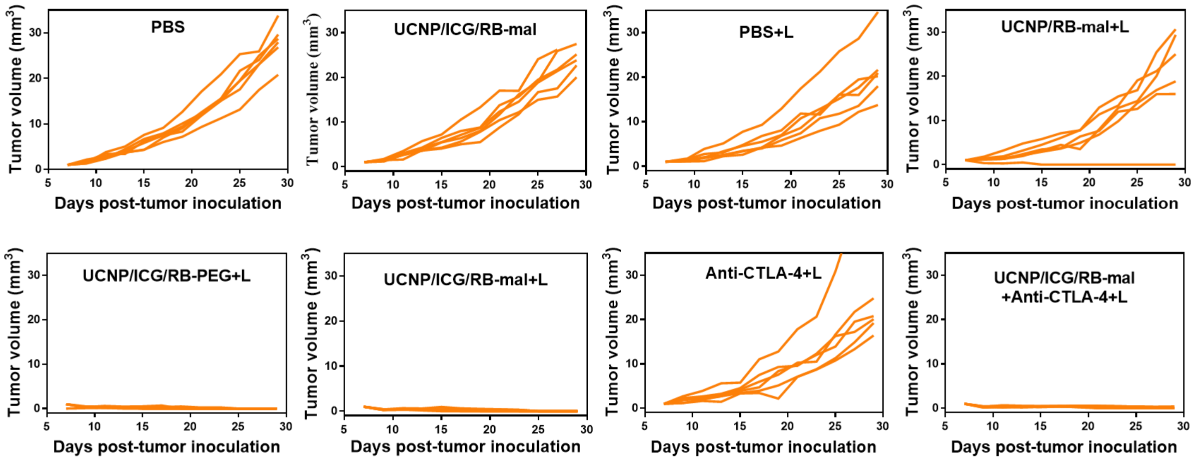

Figure S9. Growth curves of tumors in individual mice after various treatments.

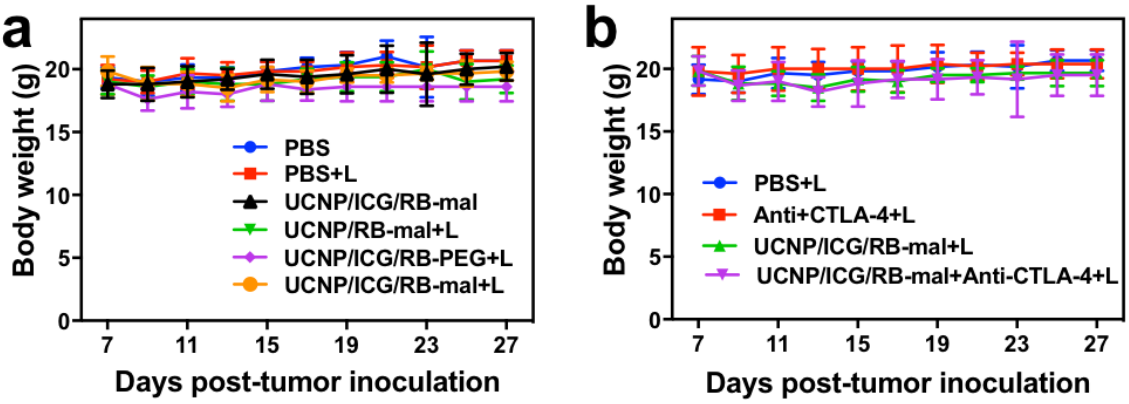

Figure S10. Average body weight of orthotopic 4T1 tumor-bearing mice of different groups. Data are expressed as means ± SD (n = 6).

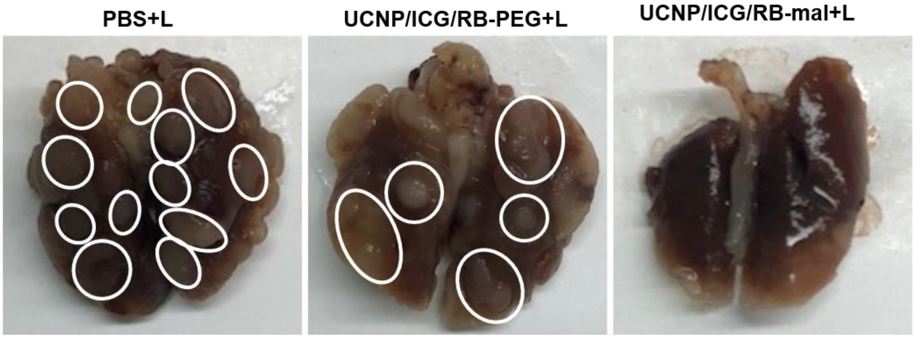

Figure S11. Photographs of metastatic nodules in lungs of mice in different groups 25 days after treatment.

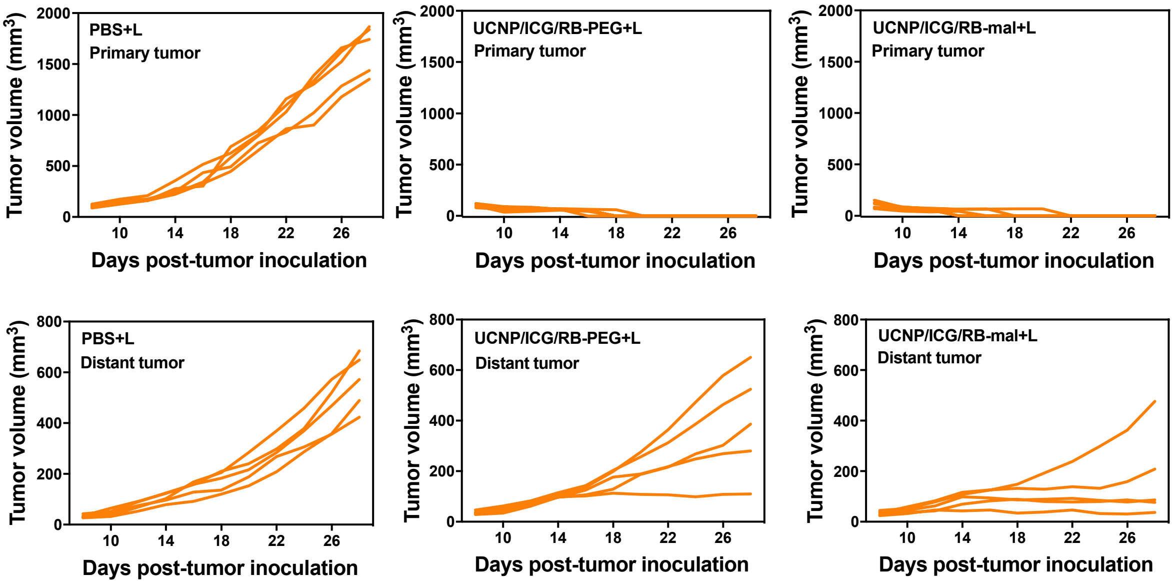

Figure S12. Growth curves of primary and distant tumors in individual mice after various treatments.

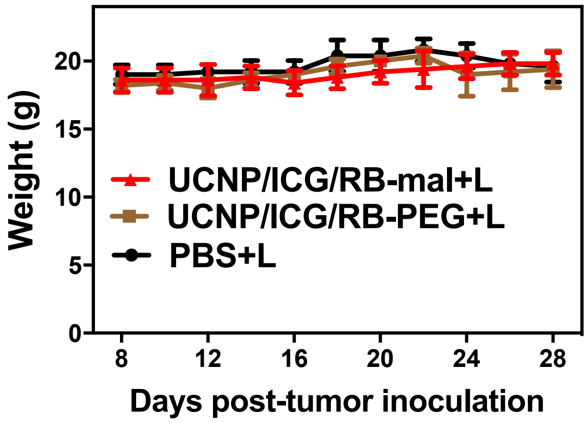

Figure S13. Average body weight of bilateral 4T1 tumor-bearing mice of different groups. Data are expressed as means ± SD (n = 5).

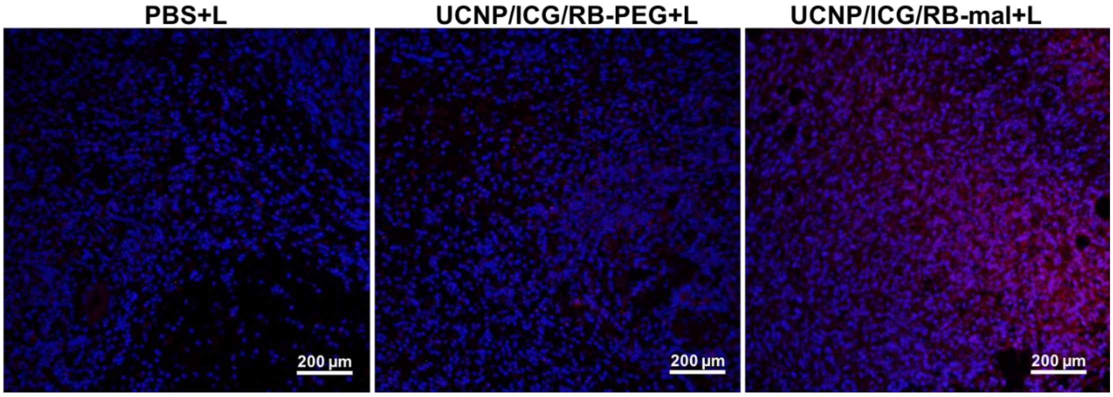

Figure S14. Immunohistochemistry staining of $CD11c^+$ dendritic cells (red) in 4T1 primary tumors 1 day after the treatments. Scale bar = 200 μM.

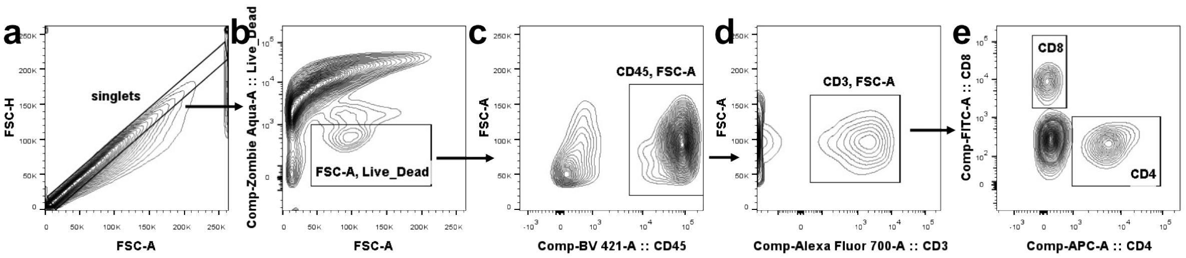

Figure S15. Gating strategy determine frequencies of T cells from distant tumors. The gating strategy was performed based on exclusion of doublets by FSC-A and FSC-H a), exclusion of dead cells b), selection of $CD45^+$ c), selection of $CD3^+$ d), and further staining using CD8 e) and CD4 e) with appropriate fluorescent dyes to select $CD8^+$ or $CD4^+$ T cells, respectively.

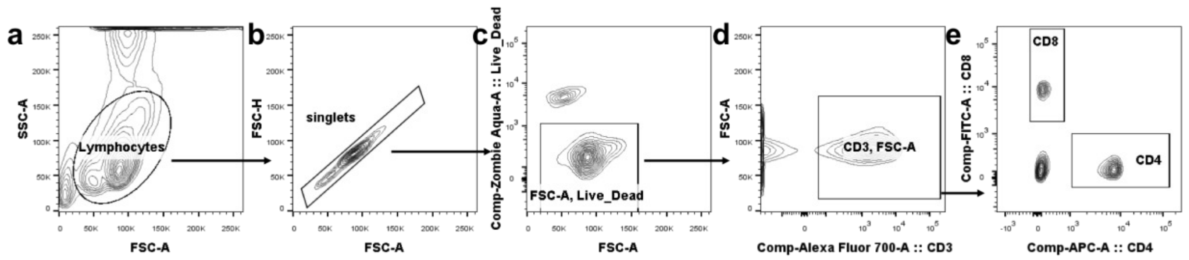

Figure S16. Gating strategy determine frequencies of T cells from spleens. The gating strategy was performed based on the right justification of the first gate a), exclusion of doublets by FSC-A and FSC-H b), exclusion of dead cells c), selection of $CD3^+$ d), and further staining using CD8 e) and CD4 e) with appropriate fluorescent dyes to select $CD8^+$ or $CD4^+$ T cells, respectively.

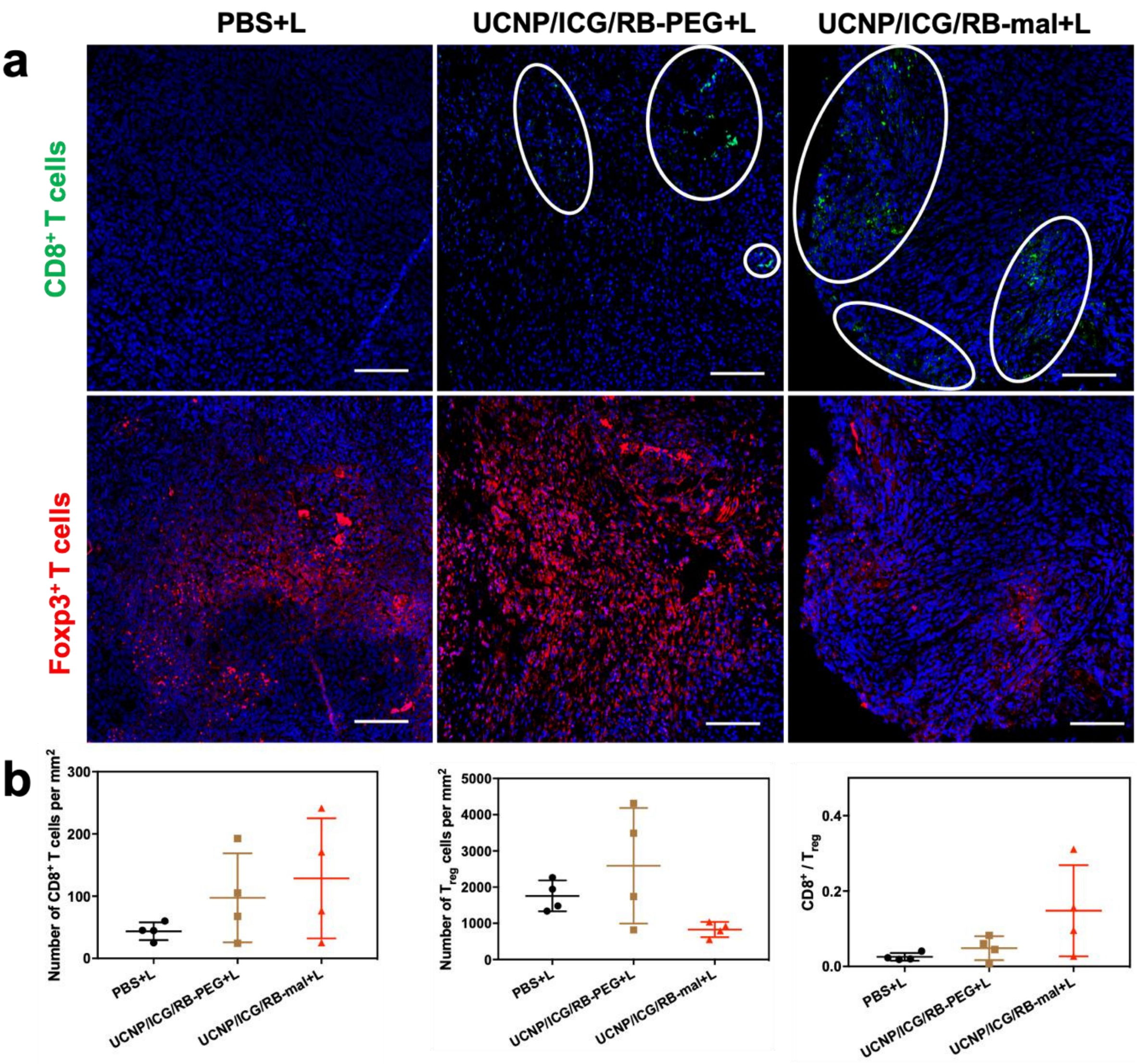

Figure S17. T cells immunofluorescence assay. a) Representative images of tumors after staining of CD8$^+$ T cells (Green) and FoXp3$^+$ T cells (T_{reg}) (Red) in 4T1 untreated second tumors 7 days after the treatments of the primary tumors. Scale bar = 200 μM. b) The densities of CD8$^+$ T cells and T_{reg} cells in the tumors. Data are expressed as means ± SD (n = 4).

Table S1. The capture of TDPAs by UCNP/ICG/RB-mal.

	Gene	Description	iBAQ
**1**	Hist2h2ab	Histone H2A type 2-B, PE=1 SV=3	145001.046
**2**	Hist2h2ac	Histone H2A type 2-C, PE=1 SV=3	25180.155
**3**	Hist1h4a,	Histone H4, PE=1 SV=2	668804.651
**4**	Hist1h2bf	Histone H2B type 1-F/J/L, PE=1 SV=2	1220846.62
**5**	Hmgb1	High mobility group protein, PE=1 SV=4	875.588785
**6**	Hspd1	60 kDa heat shock protein, PE=1 SV=1	82812.8744
**7**	Hsph1	Heat shock protein 105 kDa, PE=1 SV=2	5658.95921
**8**	Hsp90ab1	Heat shock protein HSP 90-beta, PE=1 SV=3	21224.0829
**9**	Hspa1b	Heat shock 70 kDa protein 1B, PE=1 SV=3	6718.14642
**10**	Hspa8	Heat shock cognate 71 kDa protein, PE=1 SV=1	109807.119
**11**	Hspa4l	Heat shock 70 kDa protein 4L, PE=1 SV=2	349.673031
**12**	Hsp90aa1	Heat shock protein HSP 90-alpha, PE=1 SV=4	15045.1419
**13**	Hsp90ab1	Heat shock protein HSP 90-beta, PE=1 SV=3	21224.0829
**14**	Hspd1	60 kDa heat shock protein, PE=1 SV=1	82812.8744
**15**	Trap1	Heat shock protein 75 kDa, PE=1 SV=1	43148.83
**16**	Hspa4	Heat shock 70 kDa protein 4, PE=1 SV=1	12998.5256
**17**	Hspd1	60 kDa heat shock protein, PE=1 SV=1	2155.54439
**18**	Mthfd1	C-1-tetrahydrofolate synthase, PE=1 SV=4	36050.4115
**19**	Mthfd1l	Monofunctional C1-tetrahydrofolate synthase, PE=1 SV=2	2155.54439
**20**	Mthfd1	C-1-tetrahydrofolate synthase, PE=1 SV=4	6686.73136
**21**	Actn4	Alpha-actinin-4, PE=1 SV=1	26667.0449
